# 4-Chloro­phenyl 4-chloro­benzoate

**DOI:** 10.1107/S1600536808022800

**Published:** 2008-07-23

**Authors:** B. Thimme Gowda, Sabine Foro, K. S. Babitha, Hartmut Fuess

**Affiliations:** a Department of Chemistry, Mangalore University, Mangalagangotri 574 199, Mangalore, India; b Institute of Materials Science, Darmstadt University of Technology, Petersenstrasse 23, D-64287 Darmstadt, Germany

## Abstract

The structure of the title compound (4CP4CBA), C_13_H_8_Cl_2_O_2_, resembles those of 4-methyl­phenyl 4-chloro­benzoate (4MP4CBA), 4-chloro­phenyl 4-methyl­benzoate (4CP4MBA) and 4-methyl­phenyl 4-methyl­benzoate (4MP4MBA), with similar bond parameters. The dihedral angle between the two benzene rings in 4CP4CBA is 47.98 (7)°, compared with 51.86 (4)° in 4MP4CBA, 63.89 (8)° in 4CP4MBA and 63.57 (5)° in 4MP4MBA. In the crystal structure, mol­ecules are linked into helical chains running along the *b* axis by C—H—O hydrogen bonds.

## Related literature

For related literature, see: Gowda *et al.* (2007 ▶); Gowda, Foro *et al.* (2008 ▶); Gowda, Svoboda *et al.* (2008 ▶); Nayak & Gowda (2008 ▶).
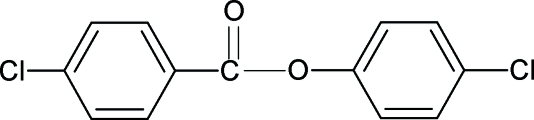


## Experimental

### 

#### Crystal data

C_13_H_8_Cl_2_O_2_
*M* *_r_* = 267.09
Monoclinic, 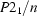

*a* = 15.370 (2) Å
*b* = 3.9528 (4) Å
*c* = 19.465 (2) Å
β = 91.804 (9)°
*V* = 1182.0 (2) Å^3^
*Z* = 4
Cu *K*α radiation
μ = 4.83 mm^−1^
*T* = 299 (2) K
0.45 × 0.23 × 0.18 mm

#### Data collection

Enraf–Nonius CAD-4 diffractometer
Absorption correction: ψ scan (North *et al.*, 1968 ▶) *T*_min_ = 0.194, *T*_max_ = 0.420
4211 measured reflections
2109 independent reflections
1724 reflections with *I* > 2σ(*I*)
*R*_int_ = 0.080
3 standard reflections frequency: 120 min intensity decay: 1.0%

#### Refinement

*R*[*F* ^2^ > 2σ(*F* ^2^)] = 0.048
*wR*(*F* ^2^) = 0.139
*S* = 1.05
2109 reflections
178 parameters
Only H-atom coordinates refined
Δρ_max_ = 0.39 e Å^−3^
Δρ_min_ = −0.33 e Å^−3^

### 

Data collection: *CAD-4-PC* (Enraf–Nonius, 1996 ▶); cell refinement: *CAD-4-PC*; data reduction: *REDU4* (Stoe & Cie, 1987 ▶); program(s) used to solve structure: *SHELXS97* (Sheldrick, 2008 ▶); program(s) used to refine structure: *SHELXL97* (Sheldrick, 2008 ▶); molecular graphics: *PLATON* (Spek, 2003 ▶); software used to prepare material for publication: *SHELXL97*.

## Supplementary Material

Crystal structure: contains datablocks I, global. DOI: 10.1107/S1600536808022800/ci2640sup1.cif

Structure factors: contains datablocks I. DOI: 10.1107/S1600536808022800/ci2640Isup2.hkl

Additional supplementary materials:  crystallographic information; 3D view; checkCIF report

## Figures and Tables

**Table 1:** Hydrogen-bond geometry (Å, °)

*D*—H⋯*A*	*D*—H	H⋯*A*	*D*⋯*A*	*D*—H⋯*A*
C5—H5⋯O2^i^	0.92 (3)	2.58 (3)	3.168 (3)	123 (2)

Symmetry code: (i) 
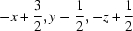
.

**Fig. 1.**
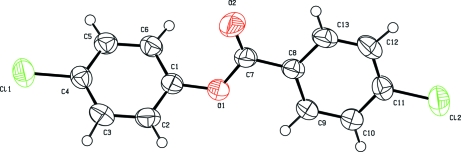
Molecular structure of the title compound, showing the atom labelling scheme. Displacement ellipsoids are drawn at the 50% probability level. H atoms are represented as small spheres of arbitrary radius.

**Fig. 2.**
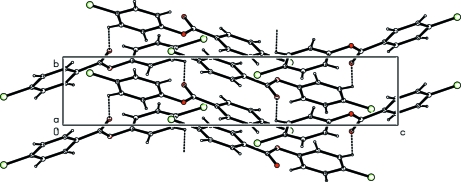
Molecular packing of the title compound.

## supplementary crystallographic information

### Comment

As part of a study of the substituent effects on the solid state structures of aryl benzoates (Gowda *et al.*, 2007; Gowda, Foro *et al.*, 2008; Gowda, Svoboda *et al.*, 2008), the crystal structure of 4-chlorophenyl 4-chlorobenzoate (4CP4CBA) has been determined. The structure (Fig. 1) is similar to those of 4-methylphenyl 4-chlorobenzoate (4MP4CBA) (Gowda, Svoboda *et al.*, 2008), 4-chlorophenyl 4-methylbenzoate (4CP4MBA) (Gowda, Foro *et al.*, 2008) and 4-methylphenyl 4-methylbenzoate (4MP4MBA) (Gowda *et al.*, 2007). The bond parameters in 4CP4CBA are similar to those in afore mentioned 4MP4CBA, 4CP4MBA, 4MP4MBA and other aryl benzoates (Gowda *et al.*, 2007; Gowda, Foro *et al.*, 2008; Gowda, Svoboda *et al.*, 2008). The dihedral angle between the benzene and benzoyl rings in 4CP4CBA is 47.98 (7)°, compared to the values of 51.86 (4)° in 4MP4CBA, 63.89 (8)° in 4CP4MBA and 63.57 (5)° in 4MP4MBA.

In the crystal structure, the molecules are linked into helical chains (Fig. 2) running along the *b* axis by C—H—O hydrogen bonds (Table 1).

### Experimental

The title compound was prepared according to a literature method (Nayak & Gowda, 2008). The purity of the compound was checked by determining its melting point. It was characterized by recording its infrared and NMR spectra (Nayak & Gowda, 2008). Single crystals of the title compound used in X-ray diffraction studies were obtained by slow evaporation of its ethanol solution.

### Refinement

H atoms were located in a difference map and their positional parameters were refined [C-H = 0.90 (3)–0.97 (3) Å] with U_iso_(H) = 1.2*U*_eq_(C).

### Crystal data

**Table:**

C_13_H_8_Cl_2_O_2_	*F*_000_ = 544
*M**_r_* = 267.09	*D*_x_ = 1.501 Mg m^−^^3^
Monoclinic, *P*2_1_/*n*	Cu *K*α radiation λ = 1.54180 Å
Hall symbol: -P 2yn	Cell parameters from 25 reflections
*a* = 15.370 (2) Å	θ = 11.5–24.3º
*b* = 3.9528 (4) Å	µ = 4.83 mm^−^^1^
*c* = 19.465 (2) Å	*T* = 299 (2) K
β = 91.804 (9)º	Rod, colourless
*V* = 1182.0 (2) Å^3^	0.45 × 0.23 × 0.18 mm
*Z* = 4	

### Data collection

**Table:**

Enraf–Nonius CAD-4 diffractometer	*R*_int_ = 0.080
Radiation source: fine-focus sealed tube	θ_max_ = 67.0º
Monochromator: graphite	θ_min_ = 3.6º
*T* = 299(2) K	*h* = −18→18
ω/2θ scans	*k* = −4→0
Absorption correction: ψ scan(North *et al.*, 1968)	*l* = −23→23
*T*_min_ = 0.194, *T*_max_ = 0.420	3 standard reflections
4211 measured reflections	every 120 min
2109 independent reflections	intensity decay: 1.0%
1724 reflections with *I* > 2σ(*I*)	

### Refinement

**Table:**

Refinement on *F*^2^	Secondary atom site location: difference Fourier map
Least-squares matrix: full	Hydrogen site location: difference Fourier map
*R*[*F*^2^ > 2σ(*F*^2^)] = 0.048	Only H-atom coordinates refined
*wR*(*F*^2^) = 0.139	*w* = 1/[σ^2^(*F*_o_^2^) + (0.0736*P*)^2^ + 0.2805*P*] where *P* = (*F*_o_^2^ + 2*F*_c_^2^)/3
*S* = 1.05	(Δ/σ)_max_ = 0.021
2109 reflections	Δρ_max_ = 0.39 e Å^−^^3^
178 parameters	Δρ_min_ = −0.33 e Å^−^^3^
Primary atom site location: structure-invariant direct methods	Extinction correction: none

### Special details

**Table:**

Geometry. All e.s.d.'s (except the e.s.d. in the dihedral angle between two l.s. planes) are estimated using the full covariance matrix. The cell e.s.d.'s are taken into account individually in the estimation of e.s.d.'s in distances, angles and torsion angles; correlations between e.s.d.'s in cell parameters are only used when they are defined by crystal symmetry. An approximate (isotropic) treatment of cell e.s.d.'s is used for estimating e.s.d.'s involving l.s. planes.
Refinement. Refinement of *F*^2^ against ALL reflections. The weighted *R*-factor *wR* and goodness of fit *S* are based on *F*^2^, conventional *R*-factors *R* are based on *F*, with *F* set to zero for negative *F*^2^. The threshold expression of *F*^2^ > σ(*F*^2^) is used only for calculating *R*-factors(gt) *etc*. and is not relevant to the choice of reflections for refinement. *R*-factors based on *F*^2^ are statistically about twice as large as those based on *F*, and *R*- factors based on ALL data will be even larger.

### Fractional atomic coordinates and isotropic or equivalent isotropic displacement parameters (Å^2^)

**Table:**

	*x*	*y*	*z*	*U*_iso_*/*U*_eq_	
Cl1	0.49228 (5)	0.7640 (2)	0.08027 (4)	0.0690 (3)	
Cl2	0.72347 (5)	−0.0839 (2)	0.67678 (3)	0.0704 (3)	
O1	0.59363 (10)	0.3353 (5)	0.36024 (9)	0.0529 (5)	
O2	0.72448 (12)	0.5904 (7)	0.36130 (10)	0.0737 (7)	
C1	0.57335 (13)	0.4453 (6)	0.29332 (12)	0.0444 (5)	
C2	0.49523 (15)	0.6088 (7)	0.28247 (14)	0.0518 (6)	
H2	0.4603 (19)	0.657 (8)	0.3193 (16)	0.062*	
C3	0.46945 (15)	0.7062 (7)	0.21667 (15)	0.0546 (6)	
H3	0.4132 (19)	0.820 (9)	0.2133 (15)	0.066*	
C4	0.52292 (14)	0.6377 (6)	0.16294 (13)	0.0469 (5)	
C5	0.60108 (15)	0.4709 (7)	0.17399 (13)	0.0492 (6)	
H5	0.6337 (18)	0.438 (8)	0.1358 (16)	0.059*	
C6	0.62658 (14)	0.3724 (7)	0.23892 (13)	0.0488 (6)	
H6	0.6762 (19)	0.246 (8)	0.2482 (15)	0.059*	
C7	0.67225 (14)	0.4158 (7)	0.38938 (12)	0.0482 (6)	
C8	0.68345 (13)	0.2812 (6)	0.45961 (12)	0.0452 (5)	
C9	0.61746 (15)	0.1152 (8)	0.49299 (14)	0.0530 (6)	
H9	0.5619 (19)	0.089 (8)	0.4691 (16)	0.064*	
C10	0.62957 (16)	0.0048 (8)	0.55961 (13)	0.0538 (6)	
H10	0.584 (2)	−0.108 (8)	0.5807 (16)	0.065*	
C11	0.70901 (15)	0.0570 (7)	0.59276 (13)	0.0494 (6)	
C12	0.77573 (16)	0.2151 (8)	0.56050 (15)	0.0579 (7)	
H12	0.826 (2)	0.225 (9)	0.5856 (17)	0.069*	
C13	0.76306 (14)	0.3276 (8)	0.49402 (14)	0.0534 (6)	
H13	0.8067 (19)	0.428 (9)	0.4718 (16)	0.064*	

### Atomic displacement parameters (Å^2^)

**Table:**

	*U*^11^	*U*^22^	*U*^33^	*U*^12^	*U*^13^	*U*^23^
Cl1	0.0694 (4)	0.0793 (5)	0.0574 (4)	0.0037 (3)	−0.0138 (3)	0.0097 (4)
Cl2	0.0777 (5)	0.0819 (6)	0.0507 (4)	0.0114 (4)	−0.0142 (3)	0.0012 (3)
O1	0.0419 (8)	0.0685 (12)	0.0480 (9)	−0.0142 (8)	−0.0015 (6)	0.0071 (9)
O2	0.0623 (11)	0.0998 (18)	0.0589 (11)	−0.0387 (11)	0.0032 (9)	0.0064 (11)
C1	0.0394 (10)	0.0469 (13)	0.0469 (12)	−0.0087 (9)	0.0002 (9)	−0.0007 (11)
C2	0.0404 (11)	0.0604 (16)	0.0553 (14)	−0.0009 (10)	0.0094 (10)	−0.0063 (12)
C3	0.0395 (11)	0.0581 (15)	0.0659 (16)	0.0061 (11)	−0.0023 (10)	−0.0024 (13)
C4	0.0447 (11)	0.0462 (13)	0.0492 (12)	−0.0044 (10)	−0.0046 (9)	0.0006 (11)
C5	0.0451 (11)	0.0537 (14)	0.0491 (13)	0.0034 (10)	0.0054 (10)	−0.0044 (12)
C6	0.0393 (11)	0.0553 (14)	0.0518 (13)	0.0056 (10)	0.0007 (9)	−0.0009 (12)
C7	0.0378 (10)	0.0605 (15)	0.0463 (12)	−0.0070 (10)	0.0038 (9)	−0.0042 (11)
C8	0.0367 (10)	0.0513 (13)	0.0477 (13)	−0.0032 (9)	0.0027 (9)	−0.0065 (11)
C9	0.0368 (10)	0.0715 (17)	0.0504 (13)	−0.0101 (11)	−0.0043 (9)	0.0033 (12)
C10	0.0428 (11)	0.0678 (16)	0.0507 (13)	−0.0070 (12)	0.0003 (10)	0.0049 (13)
C11	0.0514 (12)	0.0502 (14)	0.0460 (12)	0.0065 (10)	−0.0064 (10)	−0.0059 (11)
C12	0.0413 (11)	0.0657 (17)	0.0659 (16)	0.0002 (11)	−0.0123 (11)	−0.0106 (14)
C13	0.0356 (10)	0.0625 (16)	0.0622 (15)	−0.0079 (11)	0.0026 (10)	−0.0057 (13)

### Geometric parameters (Å, °)

**Table:**

Cl1—C4	1.736 (3)	C5—H5	0.92 (3)
Cl2—C11	1.735 (3)	C6—H6	0.93 (3)
O1—C7	1.356 (3)	C7—C8	1.472 (3)
O1—C1	1.399 (3)	C8—C9	1.387 (3)
O2—C7	1.203 (3)	C8—C13	1.389 (3)
C1—C2	1.374 (3)	C9—C10	1.375 (4)
C1—C6	1.389 (3)	C9—H9	0.96 (3)
C2—C3	1.383 (4)	C10—C11	1.379 (3)
C2—H2	0.93 (3)	C10—H10	0.93 (3)
C3—C4	1.377 (4)	C11—C12	1.370 (4)
C3—H3	0.97 (3)	C12—C13	1.376 (4)
C4—C5	1.381 (3)	C12—H12	0.90 (3)
C5—C6	1.368 (4)	C13—H13	0.90 (3)
			
C7—O1—C1	119.07 (17)	O2—C7—C8	124.7 (2)
C2—C1—C6	120.9 (2)	O1—C7—C8	112.34 (19)
C2—C1—O1	117.2 (2)	C9—C8—C13	118.9 (2)
C6—C1—O1	121.7 (2)	C9—C8—C7	122.7 (2)
C1—C2—C3	119.8 (2)	C13—C8—C7	118.4 (2)
C1—C2—H2	120.1 (19)	C10—C9—C8	120.8 (2)
C3—C2—H2	120.1 (19)	C10—C9—H9	120.8 (18)
C4—C3—C2	119.2 (2)	C8—C9—H9	118.3 (18)
C4—C3—H3	126.1 (17)	C11—C10—C9	119.0 (2)
C2—C3—H3	114.7 (17)	C11—C10—H10	122 (2)
C3—C4—C5	120.8 (2)	C9—C10—H10	119 (2)
C3—C4—Cl1	119.77 (19)	C12—C11—C10	121.2 (2)
C5—C4—Cl1	119.41 (19)	C12—C11—Cl2	120.19 (19)
C6—C5—C4	120.1 (2)	C10—C11—Cl2	118.6 (2)
C6—C5—H5	124.0 (19)	C11—C12—C13	119.5 (2)
C4—C5—H5	115.8 (19)	C11—C12—H12	114 (2)
C5—C6—C1	119.1 (2)	C13—C12—H12	126 (2)
C5—C6—H6	123.0 (18)	C12—C13—C8	120.5 (2)
C1—C6—H6	117.8 (18)	C12—C13—H13	121 (2)
O2—C7—O1	122.9 (2)	C8—C13—H13	119 (2)
			
C7—O1—C1—C2	−129.5 (3)	O2—C7—C8—C9	172.1 (3)
C7—O1—C1—C6	55.0 (3)	O1—C7—C8—C9	−4.8 (4)
C6—C1—C2—C3	−0.8 (4)	O2—C7—C8—C13	−6.9 (4)
O1—C1—C2—C3	−176.3 (2)	O1—C7—C8—C13	176.2 (2)
C1—C2—C3—C4	−0.1 (4)	C13—C8—C9—C10	1.5 (4)
C2—C3—C4—C5	0.7 (4)	C7—C8—C9—C10	−177.5 (3)
C2—C3—C4—Cl1	−179.0 (2)	C8—C9—C10—C11	−0.9 (4)
C3—C4—C5—C6	−0.3 (4)	C9—C10—C11—C12	−0.2 (4)
Cl1—C4—C5—C6	179.4 (2)	C9—C10—C11—Cl2	−179.9 (2)
C4—C5—C6—C1	−0.6 (4)	C10—C11—C12—C13	0.7 (4)
C2—C1—C6—C5	1.1 (4)	Cl2—C11—C12—C13	−179.5 (2)
O1—C1—C6—C5	176.5 (2)	C11—C12—C13—C8	−0.1 (4)
C1—O1—C7—O2	3.2 (4)	C9—C8—C13—C12	−1.0 (4)
C1—O1—C7—C8	−179.9 (2)	C7—C8—C13—C12	178.0 (3)

### Hydrogen-bond geometry (Å, °)

**Table:**

*D*—H···*A*	*D*—H	H···*A*	*D*···*A*	*D*—H···*A*
C5—H5···O2^i^	0.92 (3)	2.58 (3)	3.168 (3)	123 (2)

Symmetry codes: (i) −*x*+3/2, *y*−1/2, −*z*+1/2.
